# Analysis of Primary Graft Dysfunction (PGD) Risk Factors in Lung Transplantation (LuTx) Patients

**DOI:** 10.3390/clinpract14040127

**Published:** 2024-08-15

**Authors:** Michał Jan Kubisa, Małgorzata Edyta Wojtyś, Piotr Lisowski, Dawid Kordykiewicz, Maria Piotrowska, Janusz Wójcik, Jarosław Pieróg, Krzysztof Safranow, Tomasz Grodzki, Bartosz Kubisa

**Affiliations:** 1 Departament of Orthopaedic Surgery and Traumatology, Carolina Hospital Luxmed, 02-757 Warsaw, Poland; 2 Department of Thoracic Surgery and Transplantation, Pomeranian Medical University, 70-880 Szczecin, Poland; 3 Department of Biochemistry and Medical Chemistry, Pomeranian Medical University, 70-111 Szczecin, Poland; 4 Department of Cardiac, Thoracic and Transplantation Surgery, Warsaw Medical University, 02-097 Warsaw, Poland

**Keywords:** PGD, lung transplantation, CVP

## Abstract

Background: Primary graft dysfunction (PGD) is a form of acute lung injury (ALI) that occurs within 72 h after lung transplantation (LuTx) and is the most common early complication of the procedure. PGD is diagnosed and graded based on the ratio of the partial pressure of arterial oxygen to the fraction of inspired oxygen and chest X-ray results. PGD grade 3 increases recipient mortality and the chance of chronic lung allograft dysfunction (CLAD). Method: The aim of this retrospective study was to identify new PGD risk factors. The inclusion criteria were met by 59 patients, who all received transplants at the same center between 2010 and 2018. Donor data were taken from records provided by the Polish National Registry of Transplantation and analyzed in three variants: PGD 1–3 vs. PGD 0, PGD 3 vs. PGD 0 and PGD 3 vs. PGD 0–2. Results: A multiple-factor logistic regression model was used to identify decreasing recipient age; higher donor BMI and higher donor central venous pressure (CVP) for the PGD (of the 1–3 grade) risk factor. Conclusions: Longer cold ischemia time (CIT) and higher donor CVP proved to be independent risk factors of PGD 3.

## 1. Introduction

Lung transplantation (LuTx) is a well-known therapeutic option used in the end stage of respiratory disease. Patients with chronic obstructive pulmonary disease (COPD), interstitial lung disease (ILD) and cystic fibrosis (CF) are most often qualified for LuTx. Pulmonary artery hypertension (PAH), non-CF bronchiectasis and lymphangioleiomyomatosis are less common qualifiers for LuTx [1]. According to the International Society for Heart and Lung Transplantation (ISHLT) 2021 guidelines, LuTx in adult patients should be considered when two criteria are met: I. a high (>50%) risk of death due to pulmonary disease within 2 years if the LuTx is not performed; and II. a high (>80%) overall chance of 5-year survival after the LuTx, from a general medical perspective, assuming correct lung graft function [2]. There are specific qualification criteria and particular disease indications required to put patients on a waiting list for LuTx [2]. Long-term survival after LuTx is shorter than that after other organ transplantation types despite the surgical technique and standards of post-operative care.

PGD is one of the most common LuTx complications and is considered a major negative prognostic factor in patients’ post-operative and long-term survival [3,4]. It is a form of acute lung injury (ALI), with a complex pathophysiology, that occurs within 72 h after transplantation and is characterized by hypoxia and pulmonary infiltrates [5]. PGD is diagnosed and graded based on the ratio of the partial pressure of arterial oxygen to the fraction of inspired oxygen and chest X-ray results according to the definition by ISHLT from 2005 and modified in 2016 (Table 1) [6].

Diagnosis of PGD is possible after the exclusion of cardiogenic pulmonary edema, pneumonia, stenosis of pulmonary vein outflow, acute organ rejection and content aspiration to the lungs [3]. The necessity of treatment with extracorporeal membrane oxygenation (ECMO) or ventilation of FiO_2_ > 0.5 is also a factor that can be used to diagnose PGD grade 3 [6,7]. This grade, which is the most severe, has varied from study to study and ranged from 10 to 30% [8,9,10,11,12].

PGD risk factors are divided into three groups: donor-dependent, recipient-dependent and perioperative. These factors are listed in Table 2.

With changes in organ preservation strategies, intraoperative support, and perioperative care, some previously recognized factors for PGD development—such as donor age, single lung transplantation, and intraoperative use of CBP—are becoming less significant [13,18,20].

PGD complications are divided into early and long-term categories. They can lead to, e.g., the need for respiratory support, treatment in the ICU and increases in the total cost of treatment [10,24]. Furthermore, patients with PGD 3 have higher mortality within 1 year after LuTx (PGD 3 vs. no PGD: 64.9% vs. 20.4%, respectively) [11]; a shorter life median (the life median of PGD 3 patients vs. patients with PGD 1 or 2: 4.6 vs. 7.5 years, respectively) [13] and a statistically significantly shorter distance in the “6 min walking test” than that covered by patients without PGD 3 [24]. The knowledge of new factors will enable better risk assessment of PGD occurrence, optimization of the treatment strategy in patients after transplantation and the implementation of prevention and insightful observation of patients in risk groups. The aim of this study was to identify unknown donor- and recipient-dependent factors that could potentially lead to the development of PGD, most importantly, PGD 3.

## 2. Materials and Methods

### 2.1. Population

**Figure 1:**
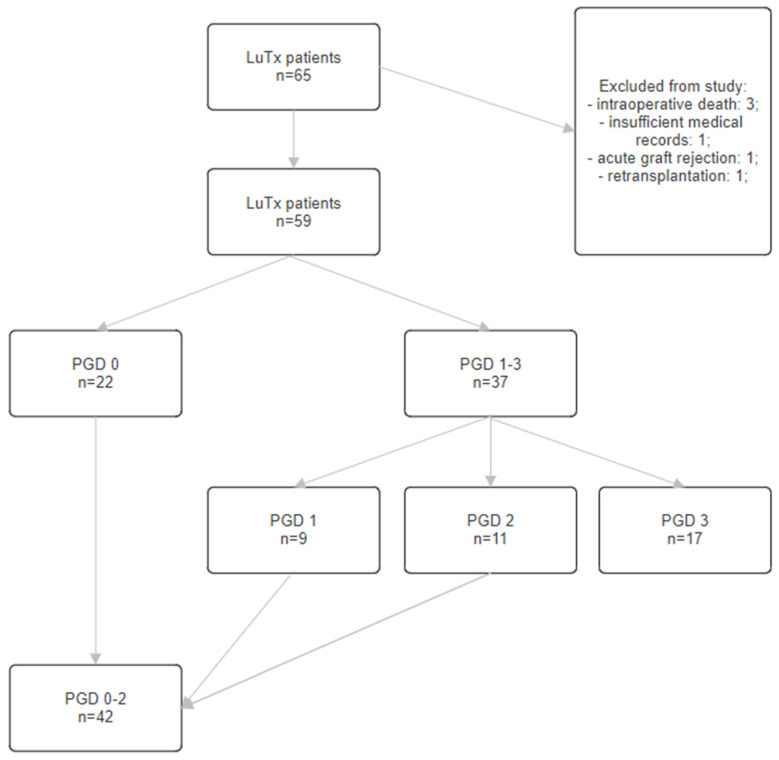
Flowchart diagram of the study cohort. LuTx—lung transplantation, PGD—primary graft dysfunction.

The data of 65 patients and their corresponding donors, who underwent LuTx at the Department of Thoracic Surgery and Lung Transplantation of the Pomeranian Medical University in Szczecin between 2010 and 2018, were analyzed (Figure 1). Out of this initial group, 6 patients were excluded. This study included 59 patients: 22 without PGD and 37 with PGD (PaO_2_/FiO_2_ <200, <300 or >300; radiological symptoms of pulmonary edema; required ECMO support due to non-cardiogenic pulmonary edema; required respiratory support with FiO_2_ >0.5). The patients with PGD were divided into 3 groups based on the grade of advancement of dysfunction: PGD 1 (9 patients), PGD 2 (11 patients) and PGD 3 (17 patients).

All patients included in this study were divided into four groups: PGD 0—patients without PGD; PGD 1–3—all patients with PGD in all grades; PGD 3—patients with grade 3 PGD; and PGD 0–2—all patients without PGD and all patients with grade 1 and 2 PGD. Data were analyzed in three variants: PGD 1–3 vs. PGD 0, PGD 3 vs. PGD 0 and PGD 3 vs. PGD 0–2 (Table 3 and Table 4).

### 2.2. LuTx Recipient Data

Arterial blood gas was taken from the radial artery of each patient 6, 24, 48 and 72 h post-operation, which is in accordance with other studies of and guidelines for PGD [6]. A chest X-ray was taken each day after the LuTx. Information about amounts of units of blood, units of fresh frozen plasma and fluids administered, and the necessity of cardiopulmonary bypass (CPB) or ECMO use during the operation were taken from surgical history records. Cold ischemia time (CIT) was presented as the time of lung implantation. Biometric data and medical history were taken from the recipients’ medical records.

### 2.3. LuTx Donor Data

The donors’ data were obtained from the Polish National Registry of Transplantation records, provided by POLTRANSPLANT. Clinical data including central venous pressure (CVP), and the results of biochemical tests were available from each donor’s primary care facility and were measured within 24 h before organ harvesting.

### 2.4. Statistical Analysis

This was a single-center study, and all patients who underwent LuTx in our department between 2010 and 2018 were included. The quantitative variables underwent a Shapiro–Wilk test for normality of distribution. Because most of these variables showed distributions that notably deviated from normalcy, they were analyzed with the help of a non-parametric Mann–Whitney U Test to see how significant the differences were between the control and study groups. The values of the quantitative variables were presented as averages ± standard deviation and as medians (lower quartile–upper quartile). Qualitative variables were compared between the groups using the chi-square test or the two-sided exact Fisher test.

In the multiple-factor analysis, in order to determine independent risk factors for PGD, logistic regression was used. Due to the small number of patients included in this study, only the 4–5 independent variables that showed the strongest relations to the development of PGD or PGD 3 in the single-factor analysis were included in the logistic regression model. Table 3 shows the final model. The results therein are presented as odds ratios (ORs) and 95% confidence intervals (95% CIs).

The threshold for statistical significance was set at *p* < 0.05. Any necessary calculation for statistical parameters was performed with Statistica 13 software.

## 3. Results

### 3.1. Variant 1—PGD 1–3 Group vs. PGD 0 Group

The first variant compared the PGD 1–3 group (n = 37) to the PGD 0 group (n = 22; Table 5 and Table 6).

**Figure 2:**
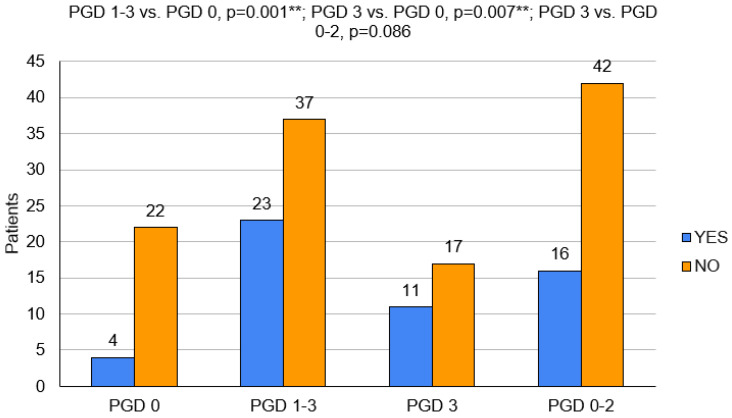
Use of CS during LuTx in particular groups (PGD 1–3 vs. PGD 0, *p* = 0.001; PGD 3 vs. PGD 0, *p* = 0.007; PGD 3 vs. PGD 0–2, *p* = 0.086)., PGD—primary graft dysfunction. **—*p* < 0.01.

In the population with PGD, statistically significant older ages (56.5 (±7.4) vs. 46.1 (±14.1); *p* = 0.009), more frequent occurrences of AB blood (*p* = 0.016) and mismatches in the blood groups (*p* = 0.048) were observed. These patients had more frequent double LuTx (*p* = 0.039) and required intraoperative circulatory support (*p* = 0.001), including ECMO (*p* = 0.026) (Figure 2).

**Figure 3:**
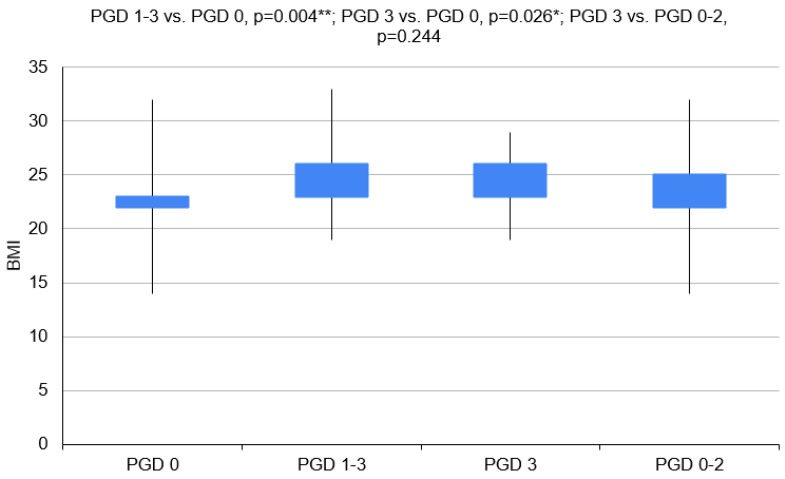
Body mass indexes of donors in particular groups (PGD 1–3 vs. PGD 0, *p* = 0.004; PGD 3 vs. PGD 0, *p* = 0.026; PGD 3 vs. PGD 0–2, *p* = 0.244). BMI—body mass index; PGD—primary graft dysfunction. **—*p* < 0.01, *—*p* < 0.05.

**Figure 4:**
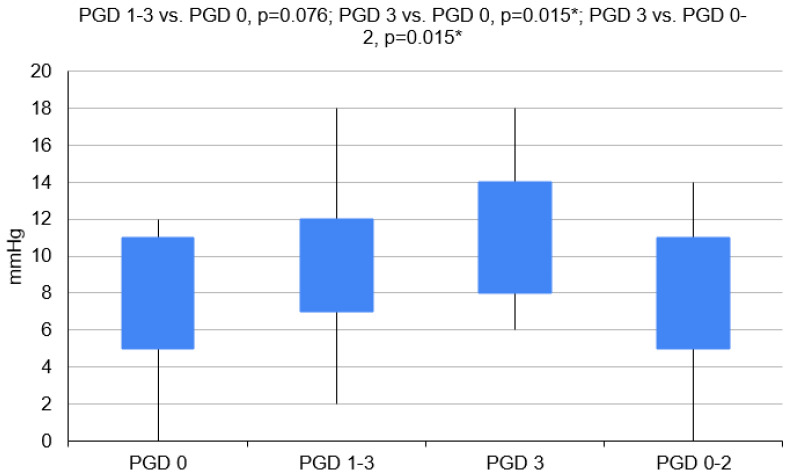
CVPs of donors in a particular group. (PGD 1–3 vs. PGD 0, *p* = 0.076; PGD 3 vs. PGD 0, *p* = 0.015; PGD 3 vs. PGD 0–2, *p* = 0.015)., PGD—primary graft dysfunction. *—*p* < 0.05.

Donors for recipients with PGD 1–3 had higher BMI values (*p* = 0.004). Patients with COPD developed PGD less often (*p* = 0.042) (Figure 3 and Figure 4).

O mismatches were excluded from this model. However, the final model included recipient age, donor BMI and CVP (Figure 4).

**Figure 5:**
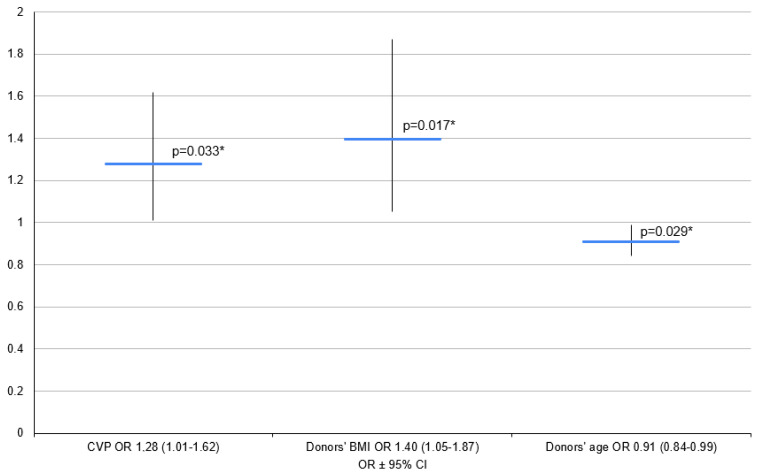
Logistic regression for models of PGD 1–3 vs. PGD 0. BMI—body mass index; CVP—central venous pressure; OR—odds ratio, 95%; 95% CI—95% confidence interval; *—*p* < 0.05.

An increase in donor age of one year was found to decrease the OR of PGD development by a multiplier of 0.91. An increase in donor BMI of 1 kg/m^2^ meant an increase in the OR of PGD development by a multiplier of 1.40, and an increase in donor CVP of 1 mm of H_2_O increased the OR of PGD development by a multiplier of 1.28 (Figure 5).

### 3.2. Variant 2—PGD 3 Group vs. PGD 0 Group

In the second variant, the study group was PGD 3 (n = 17) and the control group was PGD 0 (n = 22).

Patients with PGD grade 3 statistically significantly more often required the use of circulatory support during the operation (*p* = 0.007), the CITs of their transplanted organs were longer (*p* = 0.040), and the donors had higher CVP values (*p* = 0.015) and higher plasma creatinine concentrations (*p* = 0.017) (Figure 2 and Figure 4).

Initially, the logistic regression model included donor age, sex, CVP value, creatinine serum level and CIT. Donor age, sex and CIT were included as basic demographic parameters with proven impacts on PGD development, while donor CVP was included as a PGD parameter that was significant in the single-factor analysis.

Due to the small group size, the model did not consider circulatory support variables. The final model included donor age, sex, CVP and CIT.

**Figure 6:**
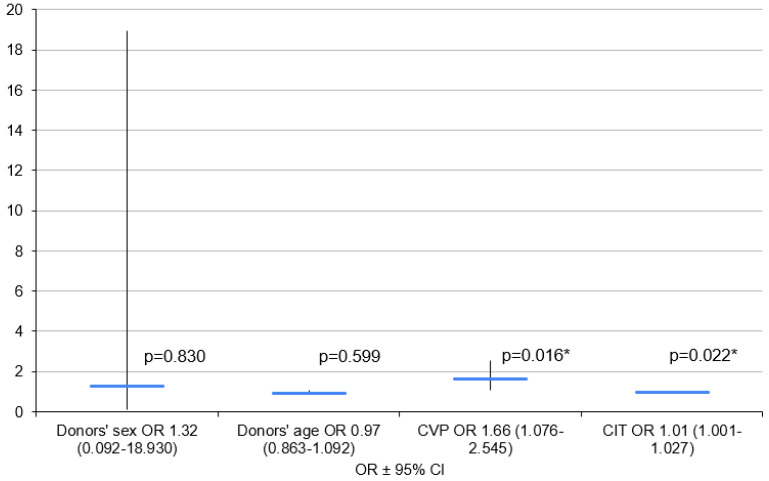
Logistic regression model for PGD 3 vs. PGD 0. CIT—cold ischemia time; CVP—central venous pressure; OR—odds ratio, 95%; 95%CI—95% confidence interval; *—*p* < 0.05.

An increase in CIT of 1 min was found to mean an increase in the OR of PGD development by a multiplier of 1.01, and an increase in donor CVP of 1 mm of H_2_O was found to mean an increase in the OR of PGD 3 development by a multiplier of 1.66 (Figure 6).

### 3.3. Variant 3—PGD 3 Group vs. PGD 0–2 Group

In the third variant, the PGD 3 group (n = 17) was compared to the PGD 0–2 group, which consisted of patients without PGD and those who developed PGD grade 1–2 (n = 42).

The donors for the PGD 3 group had statistically significantly higher values of CVP (*p* = 0.015) and creatinine serum levels (*p* = 0.008) (Table 3).

Initially, the logistic regression model included recipient age and donor age, sex, BMI and CVP. Donor age and sex were included as basic demographic parameters with known impacts on PGD development, while donor CVP and creatinine were chosen for their significant correlations with PGD in the single-factor analysis. The final logistic regression model included donor age, sex, CVP and creatinine serum level.

**Figure 7:**
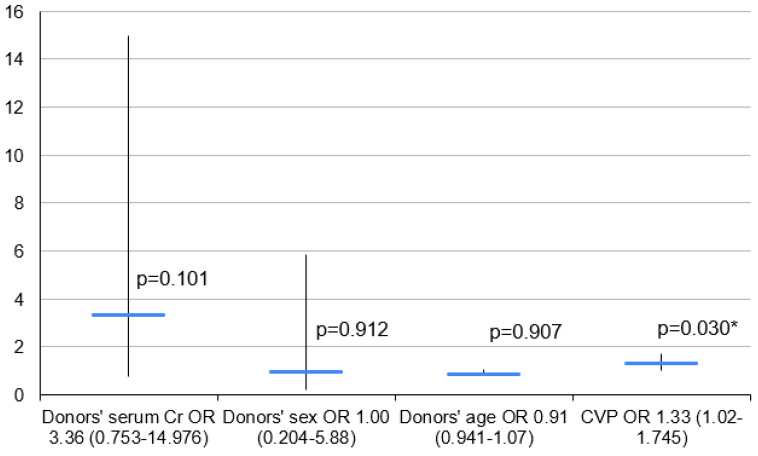
Logistic regression model for PGD 3 vs. PGD 0—2. Cr—creatinine; CVP—central venous pressure; OR—odds ratio, 95%; 95% CI—95% confidence interval; *—*p* < 0.05.

An increase in the donor CVP of 1 mm of H_2_O was shown to increase the OR of PGD 3 development by a multiplier of 1.33 (Figure 7).

## 4. Discussion

### 4.1. Key Findings and Explanations

In this study, all gathered data were used to form three independent logistic regression models, which allowed for the identification of independent PGD risk factors. The data were analyzed in two variants to account for the factors with influence on PGD 3 development. That way, the control groups consisted of PGD grade 0 patients or PGD grade 0–2 patients.

Donor CVP proved to be an independent risk factor for any PGD and PGD 3. Assuming that the physiological CVP values are 8–12 mm H_2_O [25], the CVP values of the patients with PGD grade 0 or PGD grade 1–2 were shown to be slightly below the physiological norm, while those of the PGD 3 population were at the upper limit of the norm. Abdelnour et al. stated that donor CVP below 10 mm of H_2_O allows an increased percentage of the lungs available for transplantation [26]. Pilcher et al. proved that recipient CVP of higher than 7 mm of H_2_O leads to prolonged mechanical ventilation (*p* < 0.001), increased mortality in the intensive care unit (*p* = 0.02), increased in-hospital mortality (*p* = 0.09) and an increased number of deaths within the first-month post-LuTx (*p* = 0.02) [27]. CVP is the indicator of both the intravascular fluid volume and the heart’s “preload”; higher CVP may suggest pulmonary fluid retention or even edema, which can lead to worse blood oxygenation and the consequent development of PGD. However, both authors noted that CVP is not a highly specific indicator due to various non-pulmonary reasons (e.g., cardiac diseases, and changes in fluid balance caused by resuscitation).

CIT was confirmed as another independent PGD grade 3 risk factor [28,29]. A possible explanation of the connection between PGD and prolonged CIT is increased endothelial destruction during the latter, which leads to inflammation: the core of PGD pathophysiology. Results regarding this have varied slightly among authors depending on what criteria were chosen for each particular study. According to the literature, an acceptable CIT limit is 9 h (540 min) [30]. The mean CIT for our PGD 0 population was in this range, but for the PGD 3 group, it was exceeded. Grimm et al. proved that there was no difference in PGD occurrence between CIT > 6 h and CIT < 6 h groups [31]. Similarly, Hayes et al. showed that in experienced LuTx centers, there was no difference in the number of complications between 6 h CIT and 8 h CIT groups [32]. However, in both of these studies, prolonged CIT was between 6 and 8 h. Toyoda et al. showed that 8.18 h was the cut-off value for the risk of PGD 3 [29]. In our study, the mean CIT for the PGD 3 group was over 9 h. This was also shown in a study by Kutz et al., in which the PGD risk rose with increasing CIT (3.0% for <2 h and 22.2% for >6 h) [12]. The same was found in the pediatric population, in which CIT > 6 h was linked to higher mortality [33].

Diamond et al., in a multicenter study, did not show any association between PGD and the age of the recipient [18]. Similarly, Moon et al. did not find any age difference between the PGD 0–2 and PGD 3 groups [28]. In our study, younger recipient age emerged as an independent risk factor for the development of PGD 1–3. However, contrary to the findings of Toyoda et al., we did not observe a significant association between younger recipient age and the occurrence of PGD 3 [34]. Hayes et al. noted that younger lung transplant recipients had notably worse survival rates when receiving lungs from older donors (>50 years), while older recipients showed no significant correlation of outcome with donor age [31]. In the analysis performed, older recipient age correlated with a higher probability of the occurrence of COPD. Patients with COPD have the lowest risk of PGD development [32]. However, recent studies have indicated that recipient age does not exhibit a statistically significant correlation with the development of PGD [18].

Increased recipient BMI is an independent PGD risk factor. In the literature, the BMI norm is set to be between 18.5 and 25.0 kg/m^2^. This means that the average BMI value for the PGD 0 and PGD 1–3 group fits within this norm [35,36]. The literature also features strong evidence of the increased risk of PGD in high-BMI LuTx recipients [17,18,28,29,37,38]. The main reason for this phenomenon is high leptin levels in recipients due to excess fat [39]. Leptin has a well-documented pro-inflammatory effect and a connection to PGD, consisting of leukocyte stimulation to produce cytokines [39,40]. Lederer et al. found that higher plasma leptin levels were associated with PGD [17]. However, it has not been proven that elevated leptin levels in the donor affect PGD development in the recipient. Moreover, BMI is not an ideal indicator of body composition.

### 4.2. Comparison with Similar Researches

In the literature, left ventricular diastolic dysfunction is an independent PGD risk factor [19]. This is most likely the reason for PGD development in the patients who required circulatory support. However, because these patients lacked full cardiological workups, this parameter was not included. Furthermore, the “use of circulatory support” variable for this limited patient population showed such a high statistical dependence that, if included for analysis, it would have made the other studied variables relatively insignificant. Therefore, it must be concluded that the need for additional circulatory support is a predictor of PGD development, but the identification of the specific factors directly associated with it is beyond the scope of this paper.

Due to the limited number of patients included in this study, the following potential risk factors were excluded from the analysis: bilateral lung transplantation (BLuTx), ABO mismatches and recipient serum creatinine levels. The main reason for this is that with 65 patients enrolled, these parameters showed little to no statistical significance.

### 4.3. Strengths and Limitations

The most significant limitation of this study was the small size of the studied population, which did not allow for the inclusion of more variables in the multiple-factor analysis. The next most significant problem was a lack of some data in the donor’s medical records.

Furthermore, the measurable donor parameters, e.g., CVP, Cr and INR, were obtained within a 24 h period prior to lung harvest, which means that the final values could potentially vary from the ones analyzed in this paper.

In the case of the recipients, the biggest challenge was differentiating between PGD and pneumonia.

Another problem was the cohort effect. The patient data were gathered between 2010 and 2018. Over the course of those 8 years, many advancements were introduced to various aspects of LuTx, e.g., the improvement of the surgical technique and better qualifying schemes for more accurate donor–recipient pairing. These could have had a significant effect on the PGD occurrence and falsified the results.

Finally, some well-documented PGD risk factors, e.g., thoracic closure time, increased FiO_2_ during the reperfusion phase and left ventricular diastolic dysfunction, were not included in this study.

### 4.4. Implications and Actions Needed

Further research on a larger population of patients should be performed in order to determine the impact of the variables that were identified in the single-factor analysis but not included in the multiple-factor analysis due to the insufficient number of patients in the studied population.

## 5. Conclusions

Long-term survival after LuTx is shorter than that after other organ transplantation types despite the surgical technique and standards of post-operative care. Knowledge of the PGD risk factors can improve the results of lung transplantation. Elevated recipient age demonstrates an inverse association with the incidence of primary graft dysfunction (PGD), whereas high donor body mass index (BMI) is positively correlated with an escalated probability of PGD development. Enhanced donor central venous pressure (CVP) is linked to an augmented overall risk of PGD, and specifically, an increased susceptibility to PGD grade 3. Prolonged cold ischemia time (CIT) exhibits a positive correlation with the risk of PGD grade 3 development.

## Figures and Tables

**Table 1:** PGD definition [6].

PGD Grade	PaO_2_/FiO_2_ Ratio	Pulmonary Edema on Chest X-ray	Other
0	Any	No	-
1	>300	Yes	-
2	200–300	Yes	-
3	<200	Yes	Or ECMO; or FiO_2_ > 0.5

ECMO—extracorporeal membrane oxygenation; FiO_2_—fraction of inspired oxygen; PaO_2_—partial pressure of arterial oxygen; PGD—primary graft dysfunction.

**Table 2:** PGD risk factors divided into groups [8,12,13,14,15,16,17,18,19,20,21,22,23].

Group	Risk Factors
Donor-Dependent	Brain injury, cardiac arrest before organ collection, alcoholism, nicotinism, donor age, female sex, classification into African-American population
Recipient-Dependent	Obesity, LuTx indicator diseases, cardiac diseases, female sex, classification into African-American population
Perioperative	Single lung transplant, prior pleurodesis, intraoperative use of CBP, extended CIT, numerous transfusions, higher FiO_2_ during the reperfusion phase, delayed thorax closure

CPB—cardiopulmonary bypass; CIT—cold ischemia time; FiO_2_—fraction of inspired oxygen; LuTx—lung transplantation.

**Table 3:** Demographic parameters of recipients in particular groups.

	PGD 0 Group n = 22			PGD 1–3 Group n = 37			PGD 3 Group n = 17			PGD 0–2 Group n = 42		
	n	Avg. ± SD	Med. ± (IQR)	n	Avg. ± SD	Med. ± (IQR)	n	Avg.± SD	Med. ± (IQR)	n	Avg. ± SD	Med. ± (IQR)
Age (Years)	22	56.5 (±7.4)	58 (56–62)	37	46.1 (±14.1)	50 (36–59)	17	45.5 ± 15.1	55 (39–59)	42	51.1 ± 12.0	56.5 (43–59)
BMI (kg/m^2^)	21	22.6 (±2.5)	23.2 (20.9–24.2)	36	21.8 (±3.6)	21.3 (19.5–24.6)	16	22.7 ± 3.9	21.4 (20.1–24.6)	41	21.9 ± 3.00	22.4 (19.9–24.2)
Sex (Female)	10/22 (45.5%)	-	-	14 (37.8%)	-	-	5 (29.4%)	-	-	19 (45.2%)	-	-

Avg.—average; BMI—body mass index; Med.—median.

**Table 4:** Demographic parameters of donors in particular groups.

	PGD 0 Group n = 22			PGD 1–3 Group n = 37			PGD 3 Group n = 17			PGD 0–2 Group n = 42		
	n	Avg. ± SD	Med. ± (IQR)	n	Avg. ± SD	Med. ± (IQR)	n	Avg.± SD	Med. ± (IQR)	n	Avg. ± SD	Med. ± (IQR)
Donor Data												
Age (Years)	22	39.8 ± 13.9	44 (33–55)	37	40.5 ± 12.4	43 (31–50)	17	41.5 ± 10.3	40 (32–48)	42	39.7 ± 13.9	44 (30–50)
Sex (Female)	13 (59.1%)	13 (59.1%)	-	15 (40.5%)	-	-	7(41.2%)	-	-	21	-	-
BMI (kg/m^2^)	22	22.5 ± 3.5	22.0 (21.5–22.6)	37	24.6 ± 3.0	24.5 (22.6–25.8)	17	24.4 ± 2.8	24.5 (22.3–25.1)	42	23.6 ± 3.6	22.6 (21.6–24.8)

Avg.—average; BMI—body mass index; Med.—median.

**Table 5:** Baseline recipient characteristics in particular groups.

	PGD 0 Group n = 22			PGD 1–3 Group n = 37			PGD 3 Group n = 17			PGD 0–2 Group n = 42		
	n	Avg. ± SD	Med. ± (IQR)	n	Avg. ± SD	Med. ± (IQR)	n	Avg.± SD	Med. ± (IQR)	n	Avg. ± SD	Med. ± (IQR)
Blood Type A	15 (68.2%)	-	-	14 (37.8%)	-	-	8 (47.1%)	-	-	21 (50%)	-	-
Blood Type B	6 (27.3%)	-	-	3 (18.9%)	-	-	3 (17.6%)	-	-	10 (23.8%)	-	-
Blood Type AB	0 (0%)	-	-	6 (16.2%)	-	-	4 (23.5%)	-	-	2 (4.8%)	-	-
Blood Type 0	1 (4.5%)	-	-	10 (27.0%)	-	-	2 (11.8%)	-	-	9 (21.4%)	-	-
RhD (+)	20 (90.9%)	-	-	29 (78.4%)	-	-	14 (82.4%)	-	-	35 (83.3%)	-	-
COPD	11 (50.0%)	-	-	11 (21.6%)	-	-	4 (23.5%)	-	-	15 (35.7%)	-	-
ILD	9 (40.1%)	-	-	9 (35.1%)	-	-	6 (35.2%)	-	-	16 (38.1%)	-	-
CF	2 (9.1%)	-	-	12 (32.4%)	-	-	5 (29.4%)	-	-	9 (21.4%)	-	-
Hypertension	6 (27.3%)	-	-	14 (37.8%)	-	-	6 (35.3%)	-	-	14 (33.3%)	-	-
CHD	0 (0.0%)	-	-	4 (10.8%)	-	-	2 (11.8%)	-	-	2 (4.8%)	-	-
Diabetes	4 (18.2%)	-	-	8 (21.6%)	-	-	5 (29.4%)	-	-	7 (16.7%)	-	-
Secondary PAH	14 (63.6%)	-	-	23 (62.2%)	-	-	11 (64.7%)	-	-	23 (54.8%)	-	-
Pregnancy History	7/10 (70.0%)	-	-	10/14 (71.4%)	-	-	4/5 (80.0%)	-	-	13/19 (68.4%)	-	-
Nicotinism >10 y	9 (40.1%)	-	-	13 (35.1%)	-	-	5 (29.4%)	-	-	17 (40.5%)	-	-
BLuTx	12 (54.5%)	-	-	30 (81.0%)	-	-	13 (76.5%)	-	-	29 (69.0%)	-	-
Circulatory Support	4 (18.2%)	-	-	23 (62.2%)	-	-	11 (64.7%)	-	-	16 (38.1%)	-	-
CPB	0 (0.0%)	-	-	5 (13.5%)	-	-	3 (17.6%)	-	-	2 (4.8%)	-	-
ECMO	4 (18.2%)	-	-	18 (48.6%)	-	-	8 (47.1%)	-	-	14 (33.3%)	-	-
CIT (min)	22	467 (±109)	485 (350–540)	35	534 (±123)	534 (440–600)	15	562 ± 118	540 (470–660)	42	490 ± 118	490 (380–580)
Intraoperative Fluids (mL)	19	2347 (±1150)	2200 (1700–2850)	34	3081 (±1753)	2450 (2000–3850)	15	2898 ± 1023	2500 (2300–3850)	38	2786 ± 1703	2275 (1700–3400)
RBCC (Units)	19	2.8 (±1.3)	2 (2–4)	34	4.4 (±3.4)	4 (2–6)	15	4.9 (±4.2)	4 (2–6)	38	3.4 ± 2.2	3 (2–4)

Avg.—average; BLuTx—bilateral lung transplantation; CHD—coronary heart disease; CIT—cold ischemia time; COPD—chronic obstructive pulmonary disease; CPB—cardiopulmonary bypass; ECMO—extracorporeal membrane oxygenation; ILD—interstitial lung disease; Med.—median; PAH—pulmonary artery hypertension; RBCC—red blood cell concentration.

**Table 6:** Baseline donor characteristics in particular groups.

	PGD 0 Group n = 22			PGD 1–3 Group n = 37			PGD 3 Group n = 17			PGD 0–2 Group n = 42		
	n	Avg. ± SD	Med. ± (IQR)	n	Avg. ± SD	Med. ± (IQR)	n	Avg.± SD	Med. ± (IQR)	n	Avg. ± SD	Med. ± (IQR)
Trauma	8 (36.3%)	8 (36.3%)	-	16 (43.2%)	-	-	5 (29.4%)	-	-	19 (45.2%)	-	-
SAH	10	10 (45.5%)	-	18 (48.6%)	-	-	9 (52.9%)	-	-	19 (45.2%)	-	-
Alcoholism	7	7 (31.8%)	-	14 (37.8%)	-	-	7 (41.2%)	-	-	14 (33.3%)	-	-
CVP (mm H_2_O)	19	7.3 (±3.5)	7 (5–11)	25	9.4 ± 3.9	9 (7–12)	11	11.1 ± 3.7	10 (8–14)	33	7.7 ± 3.5	7 (5–11)
WBCs (10^3^/mm^3^)	21	14.1 ± 5.3	12.5 (10.9–16.4)	35	14.8 ± 9.5	12.5 (9.7–18)	16	18.0 ± 12.4	15.8 (10.3–19.0)	40	13.1 ± 5.3	12.1 (10.4–15.2)
PLTs (10^3^/mm^3^)	21	161 ± 124	136 (79–175)	35	169 ± 102	160 (76–222)	16	181 ± 97	192 (76.5–270.5)	40	160 ± 116	136 (76.5–193.5)
Serum Cr (mg/dL)	22	1.24 ± 1.03	0.95 (0.73–1.30)	36	1.51 ± 1.14	1.04 (0.80–1.90)	17	1.96 ± 1.44	1.63 (1.03–2.17)	41	1.18 ± 0.84	0.89 (0.78–1.20)
INR	21	1.25 ± 0.23	1.23 (1.10–1.40)	35	1.29 ± 0.29	1.21 (1.08–1.45)	16	1.39 ± 0.35	1.29 (1.15–1.69)	40	1.23 ± 0.22	1.20 (1.08–1.37)
Sex Mismatch	7 (31.8%)	-	-	7 (29.7%)	-	-	6 (35.3%)	-	-	12/42 (28.6%)	-	-
ABO Mismatch	4 (18.2%)	-	-	17 (45.9%)	-	-	8 (47.1%)	-	-	13/42 (31.0%)	-	-
Rh Mismatch	7 (31.8%)	-	-	8 (21.6%)	-	-	2 (11.8%)	-	-	13/42 (31.0%)	-	-

Avg.—average; Cr—creatinine, CVP—central venous pressure; Med.—median; PLTs—platelets; SAH—subarachnoid hemorrhage; WBCs—white blood cells.

## Data Availability

Data available on request due to ethical reasons. Data on which the study was based can be found in the Polish National Registry of Transplantation; patient’s records are kept in the archives of the Department of Thoracic Surgery and Transplantation, Pomeranian Medical University, Szczecin, Poland.
